# Assessing COVID-19 vaccine hesitancy in Malaysian pregnant women: prevalence and influencing factors

**DOI:** 10.7717/peerj.21017

**Published:** 2026-03-30

**Authors:** Norhidayu Ginon, Zainab Mat Yudin, Wan Muhamad Amir W Ahmad, Azidah Abdul Kadir, Mohd Noor Norhayati, Erinna Mohamad Zon, Norsiah Ali, Ahmad Fithri Azam Abdul Rahman, Nur Harnani Abdullah, Nadia Hamimah Kamaludin, Norazlin Zainuddin, Asma Amaran, Rosnani Kasim, Norzarina Ireny Mohd Nazri, Punitha Arinima, Mohamad Ariff Fahmi Ahmad Zawawi

**Affiliations:** 1 School of Dental Sciences, Health Campus, Universiti Sains Malaysia, Kubang Kerian, Kelantan, Malaysia; 2 Department of Family Medicine, School of Medical Sciences, Health Campus, Universiti Sains Malaysia, Kubang Kerian, Kelantan, Malaysia; 3 Department of Obstetrics and Gynaecology, School of Medical Sciences, Health Campus, Universiti Sains Malaysia, Kubang Kerian, Kelantan, Malaysia; 4 Masjid Tanah Health Clinic, Masjid Tanah, Melaka, Malaysia; 5 Tengkera Health Clinic, Melaka Tengah, Melaka, Malaysia; 6 Luyang Health Clinic, Kota Kinabalu, Sabah, Malaysia; 7 Putatan Health Clinic, Kota Kinabalu, Sabah, Malaysia; 8 Sungai Limau Dalam Health Clinic, Yan, Kedah, Malaysia; 9 Kuala Nerang Health Clinic, Kuala Nerang, Kedah, Malaysia; 10 Bachok Health Clinic, Bachok, Kelantan, Malaysia; 11 Rantau Panjang Health Clinic, Rantau Panjang, Kelantan, Malaysia; 12 Broga Rural Clinic, Semenyih, Selangor, Malaysia; 13 Seksyen 19 Health Clinic, Shah Alam, Selangor, Malaysia

**Keywords:** COVID-19, Pregnant woman, Prevalence, Vaccination hesitancy

## Abstract

**Background:**

The World Health Organization has highlighted vaccine hesitancy as one of the top ten threats to global health, and the recent COVID-19 pandemic has demonstrated the importance of vaccination as one of the successful preventive measures, especially for high-risk groups, including pregnant women. This study aims to determine the prevalence of COVID-19 vaccine hesitancy and its influencing factors among pregnant women in Malaysia.

**Methods:**

This cross-sectional study was conducted from June 2024 to December 2024. among pregnant women at an antenatal clinic in Malaysia. Participants were selected using a non-proportionate stratified multistage cluster random sampling. The vaccine hesitancy status is measured by the Pregnancy Vaccine Hesitancy Scale (pVHS). Data were collected using a self-administered questionnaire, available through Google Forms or in printed form.

**Results:**

A total of 595 pregnant women participated (response rate: 99.2%). The prevalence of COVID-19 vaccine hesitancy was 42.5%. Vaccine hesitancy was significantly associated with incomplete or non-receipt of COVID-19 vaccination (*p* = 0.022), having family or friends who experienced severe vaccine side effects (*p* = 0.020), and lower knowledge scores regarding COVID-19 infection and vaccination (*p* < 0.001).

**Conclusion:**

These findings underscore the importance of targeted communication strategies in addressing vaccine hesitancy among pregnant women. Moreover, involving healthcare providers in disseminating accurate information and addressing patients’ concerns can be crucial in increasing vaccine acceptance among pregnant women.

## Introduction

Vaccination during pregnancy is a critical public health strategy for protecting maternal and neonatal health. During the COVID-19 pandemic, pregnant women and their neonates experienced disproportionately higher risks of morbidity and mortality, underscoring the importance of effective immunisation strategies (Gholami et al., 2023; Wei et al., 2021). Maternal immunisation reduces the risks of severe illness in mothers and provides passive immunity to infants, including protection against influenza, pertussis, and COVID-19. Vaccination is a well-established and highly effective public health intervention for the prevention of infectious diseases and achieving herd immunity within populations (Peng et al., 2016; Plotkina, 2019).

Global health authorities, including the World Health Organization (WHO) and the Centers for Disease Control and Prevention (CDC), recommended the COVID-19 vaccine as one of the vaccines that should be offered during pregnancy to reduce morbidity and mortality (US Centers for Disease Control Prevention, 2024). Evidence indicated that COVID-19 vaccination during pregnancy reduces the risk of severe maternal morbidity and adverse birth (Razzaghi et al., 2022). Despite the proven effectiveness of vaccination programmes in reducing the burden of emerging infectious diseases, including COVID-19, vaccine hesitancy has emerged as a significant barrier to achieving optimal immunisation coverage and sustaining herd immunity (Wie, Jung & Kim, 2023). Vaccine hesitancy is defined as the delay in acceptance, reluctance, or refusal of vaccination despite the availability of vaccination services (MacDonald et al., 2015) and was identified by the WHO as one of the top 10 global health threats in 2019.

Vaccine hesitancy among pregnant women is a significant public health concern, as it directly affects maternal and neonatal health outcomes. Hesitancy towards vaccine uptake varies across geographic settings, over time, and by vaccine type, influenced by factors, including such as disease, the convenience of access, and confidence in the vaccine itself (Casubhoy et al., 2024). The prevalence of vaccine hesitancy in pregnant women ranges from 43% to 55% (Ashkir et al., 2023; Firouzbakht et al., 2022; Moschese et al., 2023).

Hesitancy toward COVID-19 vaccines and trust in vaccines more generally have been shown to be significantly associated with sociodemographic factors such as age, sex, race, and education, with younger individuals and those with lower levels of education and income reporting greater hesitancy (McElfish et al., 2021). Hesitancy may arise from concerns regarding safety and effectiveness, as well as mistrust in either vaccine development or regulatory processes (Gianfredi et al., 2023). Conversely, key predictors of COVID-19 vaccine acceptance include confidence in vaccine safety and efficacy, recognition of the benefits of vaccination, concern about COVID-19 infection, adherence to public health guidelines, and trust in the healthcare system (Skjefte et al., 2021). The COVID-19 pandemic posed significant public health challenges, particularly for vulnerable populations such as pregnant women, for whom morbidity and mortality reports have increased (Allotey et al., 2020). In Malaysia, although national vaccination programmes have been widely implemented, limited evidence exists regarding the specific factors influencing COVID-19 vaccine hesitancy among pregnant women. Addressing this research gap is essential to inform targeted public health strategies, strengthen vaccine confidence, and improve maternal and neonatal outcomes. Therefore, this study aims to determine the prevalence of COVID-19 vaccine hesitancy and its influencing factors among pregnant women in Malaysia.

## Materials & Methods

### Study design and participant

**Figure 1:**
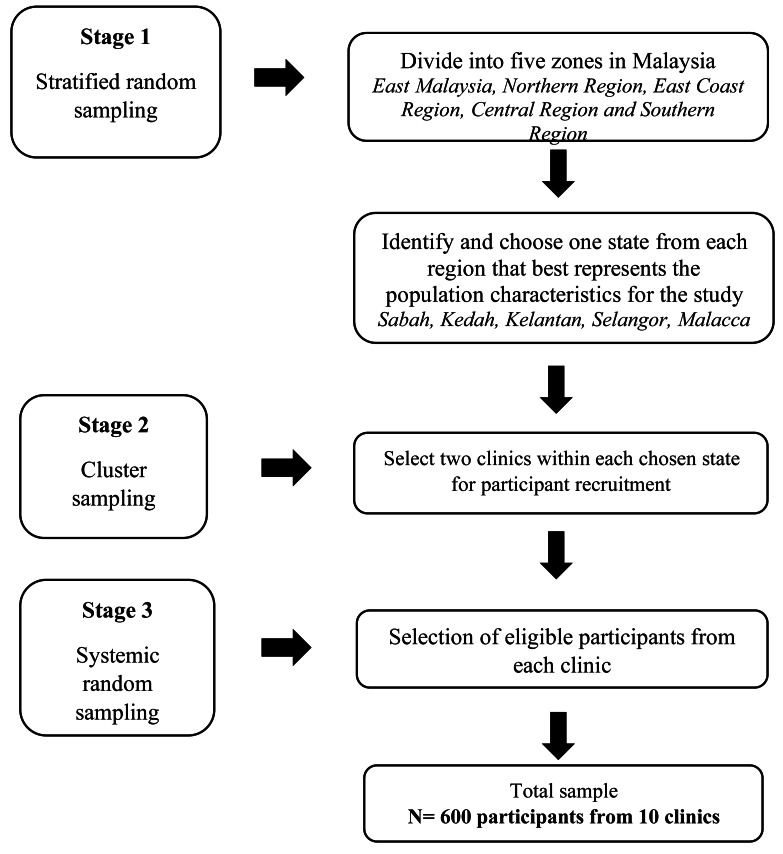
The flow of multistage sampling method.

We conducted a cross-sectional study from June 2024 to December 2024 in 10 antenatal primary care clinics across five geographic zones in Malaysia, using a multistage sampling method (Fig. 1). Malaysia was divided into five geographic zones: East Malaysia (Sabah, Sarawak, and Labuan), the Northern Region (Perlis, Kedah, Penang, and Perak), the East Coast Region (Kelantan, Terengganu, and Pahang), the Central Region (Selangor and the Federal Territories of Kuala Lumpur and Putrajaya), and the Southern Region (Negeri Sembilan, Malacca, and Johor). One state was randomly selected from each zone, followed by selecting two antenatal primary care clinics from each state, yielding 10 clinics. From each clinic, 60 pregnant women were invited to participate, yielding a total of 600 potential participants. One sub-investigator and two enumerators were appointed from each clinic to ensure the completion and timely submission of the questionnaires. The inclusion criteria include pregnant women aged 18 and above and Malaysian citizens who can read and understand Bahasa Malaysia. Pregnant women with any unstable obstetric and medical conditions, such as acute symptoms of labour, preeclampsia, pregnancy with intrauterine death, and uncontrolled hypertension, were excluded from the study. Non-proportional sampling was applied at the clinic level to ensure comparable representation across study sites despite differences in clinic attendance volumes. The sample size was calculated using a single-proportion formula, based on a previously reported study where vaccine hesitancy prevalence of 40.7% of pregnant women were hesitant toward the COVID-19 vaccine (Saitoh, Takaku & Saitoh, 2022). A precision level of 4% and a 95% confidence level were applied, with an additional 5% allowance for potential non-response, resulting in a target sample size of 600 participants.

 The study protocol was reviewed and approved by the Human Research Ethics Committee (JEPEM), Universiti Sains Malaysia (protocol number: USM/JEPeM/22050297), and Medical Research & Ethics Committee (MREC), Ministry of Health Malaysia (protocol number: 22-01456-AQA). All participants were provided with a copy of the approved informed consent form prior to completing the online or hard-copy questionnaire. Upon completion of the survey, participants received 10 Malaysian Ringgit as a token of appreciation for their time and effort.

### Study instruments and data collection

Data were collected using a self-administered questionnaire, available *via* an online Google Form (for participants with internet access) or in printed form (for participants without internet access). We developed and validated the Knowledge and Attitude toward COVID-19 Infection and Vaccination during Pregnancy Questionnaire (pKAC19) to assess knowledge (16 items) and attitude (10 items) (Mood, 2023). Content Validity, the item-level content validity index (I-CVI) is 0.96 for knowledge and 0.99 for attitude. The face Validity index (I-FVI) obtained is 0.99 for knowledge, 0.98 for attitude. Construct validity was established using exploratory factor analysis (EFA) and confirmatory factor analysis (CFA), while internal consistency reliability was assessed using Cronbach’s alpha. The EFA was conducted among 200 pregnant women attending antenatal clinics at an institutional hospital, and the CFA was conducted among 300 pregnant women attending antenatal clinics at a tertiary-level government hospital. For EFA, all items had good factor loadings with more than 0.3. The proposed model fit in all five model indices with (*χ*2 = 1,079.584 (499) *p* < 0.001; RMSEA = 0.062; CFI = 0.917; TLI = 0.857). The Cronbach alpha scores were 0.66 and 0.81 for knowledge and attitude, respectively. We used the Malay version of the Pregnancy Vaccine Hesitancy Scale (pVHS) to assess vaccine hesitancy among pregnant women. This instrument was adapted from the Adult Vaccine Hesitancy Scale (aVHS) and validated through psychometric evaluation, including exploratory factor analysis with a Cronbach’s Alpha of 0.94 (Mood et al., 2024). The authors have permission to use the instruments. The questionnaire consisted of 8 items. The scoring is based on a 5-point Likert rating scale that ranges from (1) strongly disagree, (2) disagree, (3) not sure, (4) agree, to (5) strongly agree. The results were assessed using a mean score ranging from 8 to 40. Lower scores indicate greater hesitancy toward COVID-19 vaccination among participants. We determined the cut-off for vaccine hesitancy based on the mean (Singh, 2006). Participants who score below the mean are considered more hesitant, and those who score above the mean are considered less hesitant. The questionnaire took 15–20 min to complete.

### Data analysis

All statistical analyses were performed using IBM SPSS version 29 software (IBM Corp., Armonk, NY, USA). Descriptive statistics were used to summarize the characteristics of the study population. Continuous variables were expressed as means and standard deviations, while categorical variables were presented as frequencies and percentages. The numerical variables include age (in years) and gestational age (in weeks). The categorical variables include race, religion, education level, monthly income, occupation, gravida, pre-pregnancy diseases, current disease, pre-pregnancy body mass index (BMI), history of COVID-19 infection, Family member/friend passed away due to COVID-19 infection, Family member/friend require respiratory support due to complications from COVID-19, vaccination status (1st dose/2nd dose/booster), any vaccine-related side effects, family member/friend with severe side effects and refusal of vaccination. Missing data were assessed for both the extent and the pattern of missingness. Descriptive analyses were first conducted to determine the percentage of missing values for each variable. We collapsed certain categories to accommodate small sample sizes in some groups and to ensure we had the power to assess significant associations of influencing factors. Binary logistic regression models were used to identify the factors influencing COVID-19 vaccine hesitancy. First, univariate logistic regression was performed to assess the crude association between each independent variable and the outcome. Variables with a *p*-value < 0.25 in the univariate analysis were included in the multivariate logistic regression model. The forward stepwise selection method (Likelihood Ratio) was applied to build the final multivariate model and to control for potential confounding. In the multivariate model, adjusted odds ratios (AORs) with 95% confidence intervals (CIs) were calculated to assess the strength and direction of associations between predictors and the outcome, while controlling for other covariates. A *p*-value of < 0.05 was considered statistically significant. Multicollinearity was checked using variance inflation factors (VIFs), and model fit was assessed using the Hosmer-Lemeshow goodness-of-fit test.

## Results

A total of 595 valid responses were obtained, yielding a response rate of 99.2%. Only 10% responded *via* an online Google Form. The mean age of participants was 29.90 years, SD = 5.02, with an age range of 18 to 45 years old. The mean gestational age at the time of participation was 25.8 weeks, SD = 8.63. A majority of participants were Malay (78.5%), 11.1% reported a history of pregnancy-related conditions, 46.4% reported a previous COVID-19 infection, and 48.5% had received 3 doses of the COVID-19 vaccine. This is shown in Table 1. The findings showed that vaccine hesitancy among pregnant women was more common among Malays and Muslims (both 43.3%). A higher proportion of hesitancy was observed among women with lower income (43.8%) and those who were unemployed (45.5%), suggesting that sociodemographic factors may influence attitudes during pregnancy. Overall, 42.5% of participants (*n* = 253) were classified as hesitant toward the COVID-19 vaccine. Even though almost 70% of pregnant women agreed on the need for COVID-19 vaccination to protect against severe illness, 36.7% are unsure and do not feel the need to get the COVID-19 vaccine as a preventive measure against the new variant (Table 2).

**Table 1:** The characteristics of pregnant women in the Malaysian vaccine hesitancy study (*N* = 595). Prepregnancy diseases are hypertension, diabetes mellitus type 2, asthma, autoimmune disease, anaemia, thalassemia, and breast cancer. Current diseases in pregnancy are hypertension, pre-eclampsia, eclampsia, diabetes mellitus type 2 or gestational diabetes mellitus, anaemia, thalassemia, urinary tract infection, hyperthyroid, breast cancer. There is *data missing for one case.

**Variables**	**Mean (SD)**	***n*** ** (%)**	**Less hesitant** ***n*** ** (%)**	**More hesitant** ***n*** ** (%)**
**Age (years)**	29.9 (5.02)	–		
**Race**				
Malay		467 (78.5)	265 (56.7)	202 (43.3)
Non-Malay		128 (21.5)	77 (60.2)	51(39.8)
**Religion**				
Muslim		528 (88.7)	300 (56.8)	228 (43.2)
Non-Muslim		67 (21.5)	42 (62.7)	25 (37.3)
**Educational level**				
Primary education/below		20 (3.4)	11 (55.0)	9 (45.0)
Secondary education		266 (44.7)	146 (54.9)	120 (45.1)
Tertiary education		309 (51.9)	185 (59.9)	124 (40.1)
**Estimated monthly** **household income (RM)**				
<RM5000		511 (85.9)	287 (56.2)	224 (43.8)
>RM5000		84 (14.1)	55 (65.5)	29 (34.5)
**Occupation**				
Non-working		286 (48.1)	156 (54.5)	130 (45.5)
Working		309 (51.9)	186 (60.2)	123 (39.8)
**Gravida**				
Primigravida		193 (32.4)	111 (57.5)	82 (45.2)
Multipara		329 (55.3)	191 (58.1)	138 (41.9)
Grand multipara		73 (12.3)	40 (54.8)	33 (45.2)
**Gestational age (Weeks)**	25.8 (8.63)			
^1^ **Pre-pregnancy disease**				
None		528 (88.7)	305 (57.8)	223 (42.2)
Yes		67 (11.3)	37 (55.2)	30 (44.8)
^2^ **Current disease during pregnancy**				
None		398 (66.9)	228 (57.3)	170 (42.7)
Yes		197 (33.1)	114 (57.9)	83 (42.1)
**Pre-pregnancy BMI** (*n* = 594) *				
Underweight		47 (7.9)	27 (57.4)	20 (42.6)
Normal		278 (46.7)	159 (57.2)	119 (42.8)
Overweight		148 (24.9)	80 (54.1)	68 (45.9)
Obese		121 (20.3)	76 (62.8)	45 (37.2)
**Previous history of** **COVID-19 infection**				
No		319 (53.6)	176 (55.2)	143 (44.8)
Yes		276 (46.4)	166 (60.1)	110 (39.9)
**Family members/friends passed away due to** **COVID-19 infection**				
No		522 (87.7)	294 (56.3)	228 (43.7)
Yes		73 (12.3)	48 (65.8)	25 (34.2)
**Family members/friends require respiratory support due to complications from COVID-19**				
No		530 (89.1)	299 (56.4)	231 (43.6)
Yes		65 (10.9	43 (66.2)	22 (33.8)
**Status of COVID-19 vaccination**				
1st dose only		16 (2.7)	10 (62.5)	6 (37.5)
1st & 2nd doses		285 (47.9)	147 (51.8)	137 (48.2)
1st, 2nd & booster		289 (48.5)	181 (62.4)	109 (37.6)
Not receiving		5 (0.8)	–	–
**Side effects of the** **COVID-19 vaccine**				
Mild		196 (32.9)	109 (55.6)	87 (44.4)
Severe		129 (21.7)	67 (51.9)	62 (48.1)
No side effects		270 (45.4)	166 (61.5)	104 (38.5)
**Family members/friends who refuse the COVID-19 vaccine without medical advice**				
No		538 (90.4)	307 (57.1)	231 (42.9)
Yes		57 (9.6)	35 (61.4)	22 (38.6)
**Family members/friends experienced severe side effects from the COVID-19 vaccine**				
No		532 (89.4)	314 (59.0)	218 (41.0)
Yes		63 (10.6)	28 (44.4)	35 (55.6)

**Notes.**

1 Hypertension, Diabetes, Asthma, Autoimmune disease, Anaemia, Thalassemia, Breast cancer.

2 Hypertension, Pre-eclampsia, Eclampsia, Diabetes, Anaemia, Thalassemia, Urinary Tract Infection, Hyperthyroid, Breast cancer.

* Data missing for one case.

**Table 2:** The proportion of responses in vaccine hesitancy scale items.

Item	Answer response, n (%)				
	**Strongly disagree**	**Disagree**	**Not** **sure**	**Agree**	**Strongly agree**
	(1)	(2)	(3)	(4)	(5)
**D1** The COVID-19 vaccine is essential for my health*.*	7 (1.2)	10 (1.7)	115 (19.3)	285 (47.9)	178 (29.9)
**D2** The COVID-19 vaccine is effective in reducing the severity of the infection.	4 (0.7)	10 (1.7)	105 (17.6)	294 (49.4)	182 (30.6)
**D3** The COVID-19 vaccine is important to the public health in my community.	5 (0.8)	6 (1.0)	85 (14.3)	291 (48.9)	208 (35.0)
**D4** The COVID-19 vaccine recommended by the Malaysian government is beneficial.	6 (1.0)	6 (1.0)	105 (17.6)	295 (49.6)	183 (30.8)
**D5** The information I received regarding the COVID-19 vaccine from the Malaysian Ministry of Health (MOH) is reliable.	4 (0.7)	7 (1.2)	129 (21.7)	305 (51.3)	149 (25.0)
**D6** Getting the COVID-19 vaccine shot is a great way to protect me from getting a serious illness.	5 (0.8)	14 (2.4)	118 (19.8)	297 (49.9)	161 (27.1)
**D7** In general, I agree with all the recommendations of the Ministry of Health Malaysia regarding the COVID-19 vaccine.	5 (0.8)	20 (3.4)	114 (19.2)	303 (50.9)	153 (25.7)
**D8** I need the COVID-19 vaccine as a preventive measure against the new variant.	10 (1.7)	29 (4.9)	179 (30.1)	245 (41.2)	132 (22.2)

Several factors are associated with vaccine hesitancy, as shown in Table 3. Pregnant women who had not had or had incomplete COVID-19 vaccination expressed vaccine hesitancy (*p* = 0.022) toward COVID-19 vaccination. They are significantly more likely to delay, incompletely receive, or entirely refuse vaccination. Having family members or friends who experienced severe side effects following COVID-19 vaccination was also significantly associated with vaccination hesitancy (AOR = 0.691, 95% CI [1.114–3.571]; *p* = 0.020). This suggests that indirect negative experiences, such as hearing about or witnessing adverse vaccine outcomes among close social contacts, may increase fear and mistrust toward vaccination, thereby reducing the likelihood of vaccine acceptance among pregnant women. Participants with lower knowledge scores had significantly higher odds of vaccine hesitancy (AOR = 0.855, 95% CI [0.808–0.906]; *p* < 0.001). In other words, better-informed pregnant women are more likely to accept and complete the COVID-19 vaccination. Overall, incomplete vaccination status, exposure to adverse vaccine experiences among close social contacts, and lower knowledge levels were identified as the strongest predictors of vaccine hesitancy among Malaysian pregnant women.

**Table 3:** Factors associated with vaccine hesitancy among Malaysian pregnant women.

	**Simple Logistic Regression**		**Multiple Logistic Regression**		
**Variables**	**Crude OR** **(95% CI)**	***p*** **-value**	**Adjusted OR** **(95% CI)**	**Wald** **Statistics (df)**	***p*** **-value**
**Age**	1.018 (0.985, 1.051)	0.294	1.013 (0.971, 1.058)	0.373 (1)	0.541
**Ethnicity**					
Malay	1.00				
Non-Malay	0.86 (0.583, 1.294)	0.489	0.888(0.500, 1.579)	0.164 (1)	0.686
**Religion**					
Muslim	1.00				
Non-Muslim	0.783 (0.464, 1.323)	0.361	0.834 (0.399, 1.742)	0.234 (1)	0.628
**Education**					
Primary education and below	1.00	0.472		0.142 (2)	0.931
Secondary education	1.005 (0.403, 2.504)	0.992	1.207 (0.446, 3.269)	0.138 (1)	0.711
Tertiary education	0.819 (0.330, 2.035)	0.668	1.179 (0.425, 3.277)	0.100 (1)	0.752
**Income**					
<RM5000	1.00				
>RM5000	0.676 (0.417, 1.095)	0.111	0.928 (0.526, 1.637)	0.067 (1)	0.795
**Occupation**					
Non-working	1.00				
Working	0.794 (0.573, 1.099)	0.164	0.941 (0.633, 1.398)	0.091 (1)	0.762
**Trimester in pregnancy**					
First trimester	1.00	0.364		2.410 (1)	0.300
Second trimester	0.667 (0.378, 1.177)	0.162	0.646 (0.350, 1.193)	1.949 (1)	0.163
Third trimester	0.756 (0.434, 1.318)	0.324	0.800 (0.441, 1.454)	0.534 (1)	0.465
**Gravida**					
Primigravida	1.00	0.878		0.095 (2)	0.954
Multipara	0.978 (0.683, 1.401)	0.904	0.986 (0.647, 1.502)	0.004 (1)	0.948
Grand multipara	1.117 (0.649, 1.920)	0.690	0.902 (0.445, 1.826)	0.083 (1)	0.774
**Pre-pregnancy disease** ^1^					
No	1.00				
Yes	1.109 (0.665, 1.850)	0.692	1.307 (0.587, 1.832)	0.016 (1)	0.900
**Current pregnancy disease** ^2^					
No	1.00				
Yes	0.976 (0.691, 1.380)	0.893	0.967 (0.657, 1.423)	0.030 (1)	0.863
**Pre-pregnancy BMI**					
Underweight	1.00	0.584		1.681 (3)	0.641
Normal	1.010 (0.541, 1.888)	0.974	0.963 (0.493, 1.881)	0.012 (1)	0.913
Overweight	1.147 (0.592, 2.226)	0.684	1.017 (0.494, 2.093)	0.002 (1)	0.964
Obese	0.799 (0.403, 1.587)	0.522	0.737 (0.350, 1.554)	0.642 (1)	0.423
**History of COVID-19 infection**					
No	1.00				
Yes	0.816 (0.588, 1.131)	0.221	1.017 (0.704, 1.467)	0.008 (1)	0.930
**Family/friends who died from COVID-19**					
No	1.00				
Yes	0.672 (0.402, 1.122)	0.129	0.709 (0.379, 1.328)	1.152 (1)	0.283
**Family/friends with COVID-19 complications requiring respiratory support**					
No	1.00				
Yes	0.662 (0.385, 1.138)	0.136	0.749 (0.384, 1.460)	0.720 (1)	0.396
**Received the COVID-19 vaccine**					
Incomplete/not receiving COVID-19 vaccine	1.00	0.025^*^		7.623 (2)	0.022^*^
Complete 2 doses	1.864 (0.731, 4.756)	0.193	1.385 (0.505, 3.799)	0.401 (1)	0.527
Complete 2 doses and a booster	1.204 (0.471, 3.077)	0.698	0.832 (0.303, 2.289)	0.126 (1)	0.722
**Had side effects from the COVID-19 vaccine**					
Mild	1.00	0.161		1.269 (2)	0.530
Severe	1.159 (0.742, 1.811)	0.516	1.116 (0.683, 1.823)	0.191 (1)	0.662
No side effects	0.785 (0.540, 1.140)	0.204	0.865 (0.572, 1.310)	0.467 (1)	0.494
**Family/friends who have refused the COVID-19 vaccine without a doctor’s advice**					
No	1.00	–	–	–	–
Yes	0.835 (0.477, 1.462)	0.529	0.793 (0.417, 1.508)	0.499 (1)	0.480
**Family/friends who had severe side effects from the COVID-19 vaccine**					
No	1.00	–	–	–	–
Yes	1.800 (1.064, 3.047)	0.028^*^	1.995 (1.114, 3.571)	5.405 (1)	0.020^*^
Knowledge score	0.861 (0.817, 0.907)	<0.001^*^	0.855 (0.808, 0.906)	28.856 (1)	<0.001^*^
Attitude score	1.020 (0.984, 1.057)	0.285	1.012 (0.974, 1.052)	0.370 (1)	0.543

**Notes.**

Multiple Logistic was applied.

Significant at the level of <0.05.

Log-likelihood = 749.915; Hosmer and Lemeshow Test *χ*^2^(df): 9.379(8), *p* = 0.311.

Classification Table, 64.1%.

1 Hypertension, Diabetes, Asthma, Autoimmune disease, Anaemia, Thalassemia, Breast cancer.

2 Hypertension, Pre-eclampsia, Eclampsia, Diabetes, Anaemia, Thalassemia, Urinary Tract Infection, Hyperthyroid, Breast cancer.

## Discussion

The study represents the first multicentre, multiregional investigation of COVID-19 vaccine hesitancy among pregnant women conducted in antenatal primary care clinics in Malaysia. This design enables comprehensive analysis by involving multiple independent antenatal primary care institutions across regions, ensuring a heterogeneous sample representative of diverse demographic, cultural, and socioeconomic backgrounds. The findings indicate that 42.5% of pregnant women were hesitant toward receiving the COVID-19 vaccine, a figure that reflects broader global trends observed during the pandemic. In contrast, a multicenter study across four teaching hospitals in an urban region of Malaysia reported a higher COVID-19 vaccination acceptance rate (77.1%) (Kalok et al., 2023). This suggests that vaccine attitudes differ between urban tertiary settings and primary care clinics. Similarly, a systematic review and meta-analysis reported an overall COVID-19 vaccine acceptance rate of 53.46% among pregnant women in Asian countries, namely, China, Singapore, and Vietnam (Azami et al., 2022). Higher levels of hesitancy have been reported in other settings, such as Northwest Ethiopia, where 58.6% of pregnant women declined the COVID-19 vaccine (Aynalem et al., 2022). Similarly, a study conducted in Saudi Arabia reported that although approximately 74% of pregnant women received the COVID-19 vaccine, many experienced anxiety, worry, and hesitancy regarding vaccination (Alkhalifah, AlHusseini & McGhee, 2023). These comparisons highlight that vaccine hesitancy among pregnant women remains a persistent global concern, although its magnitude varies across healthcare settings and regions. Notably, the prevalence of vaccine hesitancy in our study was lower than that reported in neighboring Southeast Asian nations. This disparity may stem from distinct healthcare communication strategies and cultural attitudes toward vaccination within Malaysia (Kwan, Loh & Looi, 2021).

Several studies have identified that the sociodemographic characteristics of vaccine-hesitant pregnant women, include younger age, unemployment status, lower education, lower economic status, and parity (Bhattacharya et al., 2022; Razzaghi et al., 2021; Yussuph et al., 2023). In our study, factors influencing vaccine hesitancy included having family or friends who had experienced severe side effects from the COVID-19 vaccine and having lower knowledge about COVID-19 infection and vaccination. Studies have shown that having knowledge about COVID-19 was a known factor to influence vaccine hesitancy in most studies (Chekol Abebe et al., 2022; Moschese et al., 2023; Sarantaki et al., 2022). Proper knowledge of the vaccine builds understanding on the importance of taking the vaccine, which in turn fosters a positive attitude towards vaccination (Zheng, Jiang & Wu, 2022). This suggests that adequate knowledge about vaccinations can help pregnant women address and overcome COVID-19 vaccine hesitancy. Conversely, another systematic review and meta-analysis observed only a weak, non-significant positive correlation between COVID-19 knowledge and vaccine acceptance (Bhattacharya et al., 2022).

A study in Pakistan found that the majority of pregnant women experience mild vaccine side effects (67.6%), predominantly after the first dose (79.4%) (Mustafa et al., 2022). Similarly, approximately 45% of pregnant women in our study had no prior experience with vaccine side effects. Our study found that prior experience with vaccine side effects was not a significant predictor of hesitancy. This suggests that while past mild reactions may cause concern, they do not necessarily deter women from future immunisation. Specifically, individuals who have experienced mild side effects from vaccines in the past may develop heightened concerns about the safety and efficacy of future immunisations; however, this does not prevent them from getting vaccinated. Family members or friends who experience significant side effects following COVID-19 vaccination, particularly those requiring medical intervention or hospitalization, can raise concerns about vaccine safety. Such incidents may amplify doubts and negatively influence an individual’s decision to receive vaccination, especially among pregnant women who already have concerns about potential risks to both their health and their unborn child. Few studies have examined the impact of social circle experiences, yet our findings highlight this as a critical determinant of vaccine hesitancy.

Incomplete vaccination or non-receipt of the COVID-19 vaccine serves as a primary behavioral indicator of vaccine hesitancy. This reluctance often stems from multifaceted barriers, including safety concerns, fear of side effects, misinformation, mistrust in healthcare systems, and a low perceived risk of infection. Specifically, fear of adverse outcomes for both mother and fetus remain a dominant driver of hesitancy among pregnant women (Miraglia del Giudice et al., 2022). Our study reinforces the idea that these behaviors are shaped by a complex interplay of individual and interpersonal factors, notably prior vaccination history, knowledge levels, and experiences within close social contacts, which were highlighted as influencing factors in this study.

These findings, however, must be interpreted within the context of certain methodological limitations. While we employed multistage sampling across five geographic zones, the reliance on 10 clinics and a final sample of 600 participants may not fully capture the heterogeneity of the pregnant women nationwide. This relatively small, clinic-based sample limits the precision of our estimates and the generalizability of the findings to the broader Malaysian context. Consequently, these results should be viewed as preliminary evidence and warrant validation through future large-scale studies. These findings provide preliminary evidence on vaccine hesitancy among pregnant women, and future studies with larger sample sizes are needed to strengthen this evidence. Despite these constraints, the study achieved a robust response rate and offers critical insights into a high-risk population. The vaccine hesitancy pattern among pregnant women during the post-pandemic era of COVID-19 is still alarming.

## Conclusions

In conclusion, this study highlights a moderately high prevalence of vaccine hesitancy among pregnant women in Malaysia, a finding that poses a significant public health challenge given the vulnerability of this population. Given that pregnancy is a period of heightened vulnerability, vaccine hesitancy in this group poses a public health challenge with implications for both maternal and neonatal outcomes. While this study provides important regional data, it must be interpreted within the context of certain limitations, including the cross-sectional design, potential social desirability bias, and a sampling strategy that may limit generalisability. Subsequent investigations should adopt longitudinal approaches to monitor changes in acceptance and quantify the impact of trust in healthcare systems. Exploring the role of trust in healthcare systems and provider recommendations may also yield valuable insights. However, this finding should still raise concerns among healthcare providers and public health officials, as even a small percentage of hesitant individuals can lead to substantial public health implications. Addressing these concerns through targeted information campaigns and emphasizing the personal and community health benefits could further enhance vaccine uptake. By focusing on personal and community benefits, public health initiatives can effectively improve uptake and protect this vulnerable population from the severe consequences of COVID-19.

##  Supplemental Information

10.7717/peerj.21017/supp-1 Supplemental Information 1 Data

10.7717/peerj.21017/supp-2 Supplemental Information 2 STROBE checklist

## Additional Information and Declarations

### Data Availability

The following information was supplied regarding data availability: The raw data is available in the Supplemental File.
